# Validation of 4D flow CMR against simultaneous invasive hemodynamic measurements: a swine study

**DOI:** 10.1007/s10554-019-01593-x

**Published:** 2019-04-08

**Authors:** Kelly Stam, Raluca G. Chelu, Nikki van der Velde, Richard van Duin, Piotr Wielopolski, Koen Nieman, Daphne Merkus, Alexander Hirsch

**Affiliations:** 1 Department of Cardiology, Thoraxcenter, Erasmus MC, University Medical Center Rotterdam, PO Box 2040, 3000 CA Rotterdam, The Netherlands; 2 Department of Radiology and Nuclear Medicine, Erasmus MC, University Medical Center Rotterdam, PO Box 2040, 3000 CA Rotterdam, The Netherlands

**Keywords:** 4D flow CMR, Flow measurement, Invasive flow probe, Swine

## Abstract

The purpose of this study was to compare invasively measured aorta flow with 2D phase contrast flow and 4D flow measurements by cardiovascular magnetic resonance (CMR) imaging in a large animal model. Nine swine (mean weight 63 ± 4 kg) were included in the study. 4D flow CMR exams were performed on a 1.5T MRI scanner. Flow measurements were performed on 4D flow images at the aortic valve level, in the ascending aorta, and main pulmonary artery. Simultaneously, flow was measured using an invasive flow probe, placed around the ascending aorta. Additionally, standard 2D phase contrast flow and 2D left ventricular (LV) volumetric data were used for comparison. The correlations of cardiac output (CO) between the invasive flow probe, and CMR modalities were strong to very strong. CO measured by 4D flow CMR correlated better with the CO measured by the invasive flow probe than 2D flow CMR flow and volumetric LV data (4D flow CMR: Spearman’s rho = 0.86 at the aortic valve level and 0.90 at the ascending aorta level; 2D flow CMR: 0.67 at aortic valve level; LV measurements: 0.77). In addition, there tended to be a correlation between mean pulmonary artery flow and aorta flow with 4D flow (Spearman’s rho = 0.65, P = 0.07), which was absent in measurements obtained with 2D flow CMR (Spearman’s rho = 0.40, P = 0.33). This study shows that aorta flow can be accurately measured by 4D flow CMR compared to simultaneously measured invasive flow. This helps to further validate the quantitative reliability of this technique.

**Electronic supplementary material:**

The online version of this article (10.1007/s10554-019-01593-x) contains supplementary material, which is available to authorized users.

## Introduction

Cardiovascular magnetic resonance (CMR) imaging has been used for flow visualization and quantification in daily clinical practice for several decades [1, 2]. CMR is the gold standard for non-invasive quantification of left and especially right heart function and shunt fraction [3, 4]. Nowadays, standard imaging protocols consists of the acquisition of cine imaging and 2D phase contrast flow measurements in multiple planes using numerous breath-holds. Especially, in complex congenital heart disease patients this can be challenging and time consuming.

A promising and rapidly evolving CMR technique is 4D flow imaging: a volumetric, free-breathing acquisition technique of flow velocity data with simultaneous assessment of anatomic structures [5]. 4D flow CMR allows flow quantification at any level within the acquired field of view and calculation of cardiac volumes and biventricular function [6, 7]. Until now, several studies have evaluated the use of 4D flow CMR for visualization and quantification of cardiac shunts [8–11]. 4D flow CMR was previously validated against echocardiography [12] and standard 2D flow CMR in humans [13, 14]. In addition, the 4D flow determined pulmonary vascular resistance was validated against in vivo measurements in a canine study [15].

In this study, we sought to validate this promising 4D flow CMR technique by direct, simultaneous comparison with 2D flow CMR and invasive flow measurements using a validated flow probe positioned around the ascending aorta in a large animal model.

## Methods

### Study design

Studies were performed in accordance with the “Guiding Principles in the Care and Use of Laboratory Animals” as approved by the Council of the American Physiological Society, and with approval of the Animal Care Committee of the Erasmus Medical Center Rotterdam (3158, 109-13-09). Nine Yorkshire x Landrace swine (5–6 months old, 21 ± 1 kg at the time of surgery, 63 ± 4 kg at the time of the CMR scan) of either sex were included in the study. The swine included in this study were part of previously published studies [16, 17].

### Chronic instrumentation of the swine

The swine were chronically catheterized for hemodynamic monitoring approximately 2 to 3 months prior to the scanning procedure. Surgical details have been extensively described previously [18]. In short, swine were sedated with an intramuscular injection of tiletamine/zolazepam (5 mg/kg), xylazine (2.25 mg/kg) and atropine (1 mg), intubated and ventilated with a mixture of O_2_ and N_2_ (1:2 v/v) to which 2% (v/v) isoflurane was added to maintain anesthesia. Under sterile conditions, the chest was opened via a left thoracotomy in the fourth intercostal space and fluid-filled polyvinylchloride catheters (B Braun Medical Inc., Bethlehem, PA, USA) were placed in the right ventricle, pulmonary artery, aorta and left atrium. A flow probe (Transonic Systems Inc., Ithaca, NY, USA) was positioned around the ascending aorta for measurement of aorta flow. The catheters were tunneled to the back and animals received analgesia (0.015 mg/kg buprenorphine i.m. and a slow-release fentanyl patch 12 μg/h for 48 h) on the day of the surgery and daily antibiotic prophylaxis (25 mg/kg amoxicillin i.v.) for 7 days.

### CMR protocol

CMR examination was performed on a 1.5T clinical scanner with a dedicated receive-only 32-channel phased-array cardiac surface coil (Discovery MR450, GE Healthcare, Milwaukee, WI, USA). The animals were sedated, and intubated as described above. Anesthesia during imaging was maintained with pentobarbital sodium (6–12 mg/kg/h). Mechanical ventilation and breath-holds were performed using a mobile ventilator (Carina™, Dräger Medical, Best, The Netherlands). Heart rate (HR) and blood pressures were monitored throughout the scan. When necessary, and always in absence of pain reflexes, muscle relaxation was achieved using pancuronium bromide (2–4 mg bolus). The image protocol consisted of 2D balanced Steady-State Free Precession (SSFP) cine imaging, 4D flow, and 2D phase contrast flow measurements. Standard long-axis and short-axis images with full left ventricular (LV) coverage were acquired using retrospectively ECG-gated SSFP cine imaging with breath-holding (FIESTA, GE Healthcare acronym). Typical scan parameters were slice thickness 6.0 mm, slice gap 0 mm, TR/TE 3.4/1.4 ms, flip angle (FA) 75°, field of view (FOV) 320 × 240 mm, acquired matrix 128 × 180, and reconstructed to a pixel size of 1.3 × 1.3 mm. The free-breathing, retrospectively ECG-gated 4D flow acquisition was performed directly after administration of a gadolinium-based contrast agent (Gadovist 1.0 mmol/mL, Bayer, Mijdrecht, The Netherlands, single dose of up to 15 mL). The 4D flow sequence has been described before [11–13], in short the sequence was prescribed in axial plane, including the entire thorax in the field of view. The k-space was filled with variable-density Poisson-disc undersampling with acceleration factors of 1.8 × 1.8 (phase × slice) and the parallel imaging algorithm used was ESPIRiT. The following imaging parameters were used: matrix 192 × 160 × 78, acquired resolution 2.1 × 1.7 × 2.8 mm, reconstructed resolution 2.1 × 1.7 × 1.4 mm, TR/TE 3.8/1.5 ms, FA 15°, views per segment 4, bandwidth 63 kHz, number of reconstructed phases 20 per cardiac cycle, and a velocity encoded value set at 250 cm/s. Scan time ranged between 5.57 and 8.51 min. Finally, one-directional through plane 2D phase contrast flow measurements of the aorta (at the level of the aortic valve or just above) and pulmonary artery were performed during an end-expiratory breath-hold. The imaging planes were planned perpendicular to the great vessels. Typical scan parameters were slice thickness 6.0 mm, matrix 256 × 166, TR/TE 4.0/2.2 ms, FA 18°, FOV 340 × 220, velocity encoding value set at 180 cm/s. The invasive flow probe was attached to the amplifier and a flow signal was obtained immediately before and after the 4D flow CMR sequence.

### Post-processing and data analysis

To assess left ventricular volumes, endocardial contours were drawn manually on end-diastolic and end-systolic 2D short axis SSFP cine images, and stroke volume and ejection fraction were calculated. No substantial mitral regurgitation was visually noted on CMR in any of the animals, therefore stroke volume (mL/beat) and cardiac output (CO, L/min) of the left ventricle were also compared to invasive measurements. To analyze the 2D phase contrast images a region of interest was manually traced around the aorta and pulmonary valve. Both 2D flow CMR and left ventricular function were analyzed with Medis software (QMass and QFlow analytical software version 8.1, Medis, Leiden, The Netherlands).

The 4D flow data were analyzed using a dedicated cloud-based post-processing software (ArterysInc, San Francisco, CA, USA). Semi-automatic eddy-current correction was applied [13]. Data were visualized, and flow quantification was performed at the level of aortic valve and at the level of the sinotubular junction/proximal part of the ascending aorta below the flow probe. Also the pulmonary flow was determined by measuring in the main pulmonary artery (MPA). For both 2D and 4D flow shunt fractions (Qp/Qs) were calculated.

Digital recording and offline analysis of HR and aorta flow were performed with MatLab (MathWorks, Natick, MA, USA) and have been described in detail elsewhere [19, 20]. Briefly, HR and aorta flow were analyzed offline using a proprietary program written in MatLab. Over at least 10 consecutive seconds, both before and after the 4D flow CMR sequence, CO and SV were determined from each individual beat and averaged. HR was calculated as the ratio of the number of beats and time. The end-diastolic time point was used to align the (average) phasic flow signals obtained with the different methods.

All analyses were performed independently from each other (2D flow and left ventricular function measurements by NvdV and AH, 4D flow CMR by RC, and invasive flow probe measurements by KS).

### Statistics

Statistical analysis was performed with SPSS software version 21 (IBM, NewYork, US) and Graphpad Prism 4 Project (San Diego, CA, US). Correlation between measurements was evaluated using Spearman’s (rho) coefficient for nonparametric data, and agreement was analyzed with Bland–Altman plots. The Spearman rho coefficient was classified as “very weak” for values of 0.00–0.19, “weak” for 0.20–0.39, “moderate” for 0.40–0.59, “strong” for 0.60–0.79 and “very strong” for 0.80–1.0 [11, 21].

## Results

**Fig. 1:**
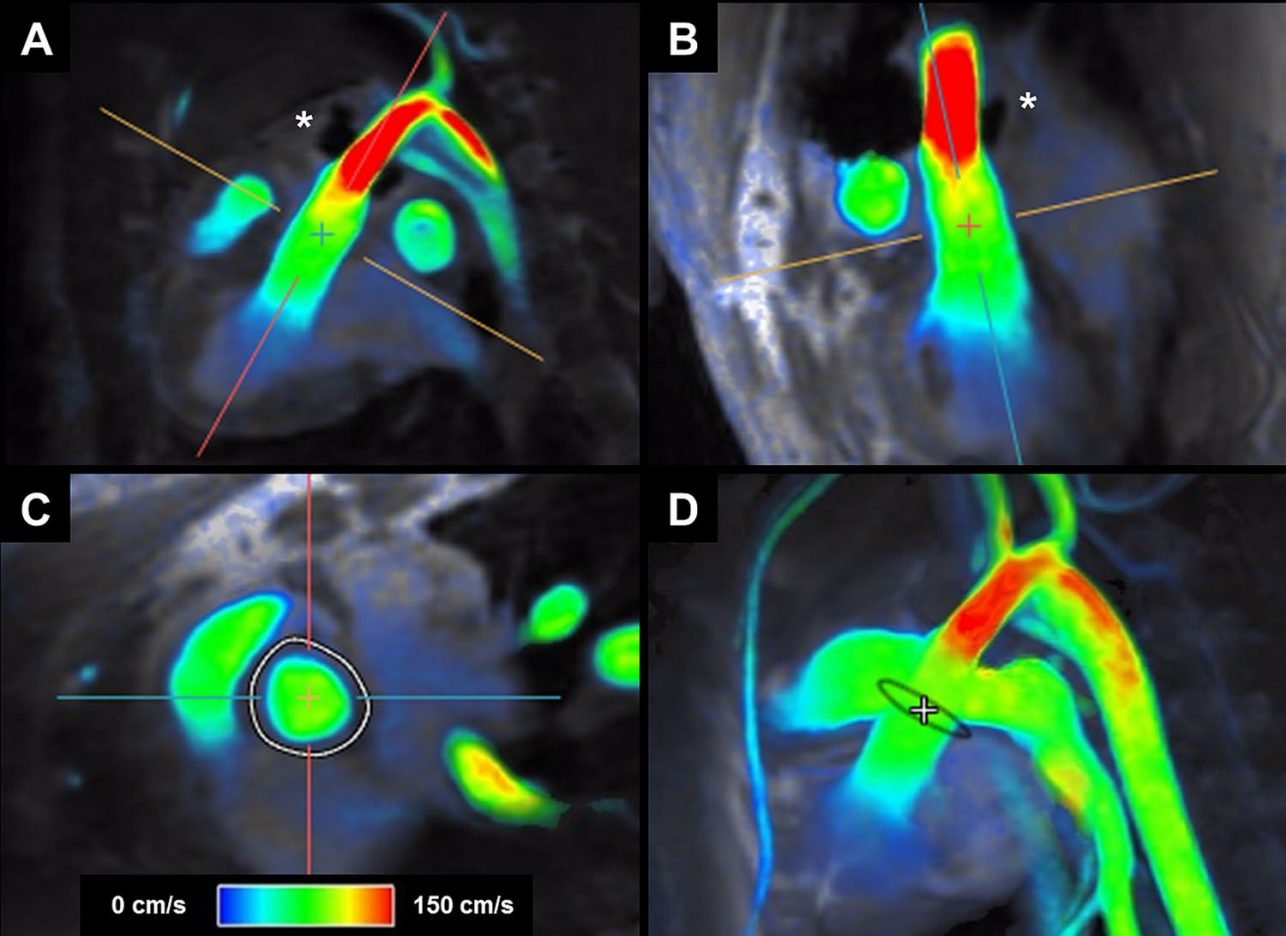
Example of 4D flow CMR images and measurement

A total of nine animals were scanned, but not all animals were included in every method of CO determination: two animals had a malfunctioning invasive flow probe, in all nine animals CO could be determined with 4D flow CMR at the level of the aortic valve, while susceptibility artefacts from the invasive flow probe precluded measurement of CO at the ascending aorta level in two animals. Finally, one animal was excluded due to technical problems for measurement of CO with 2D flow CMR as well as LV functional measurements. Typical examples of the 4D flow CMR images and measurements are shown in Fig. 1 and supplemental video’s.

**Fig. 2:**
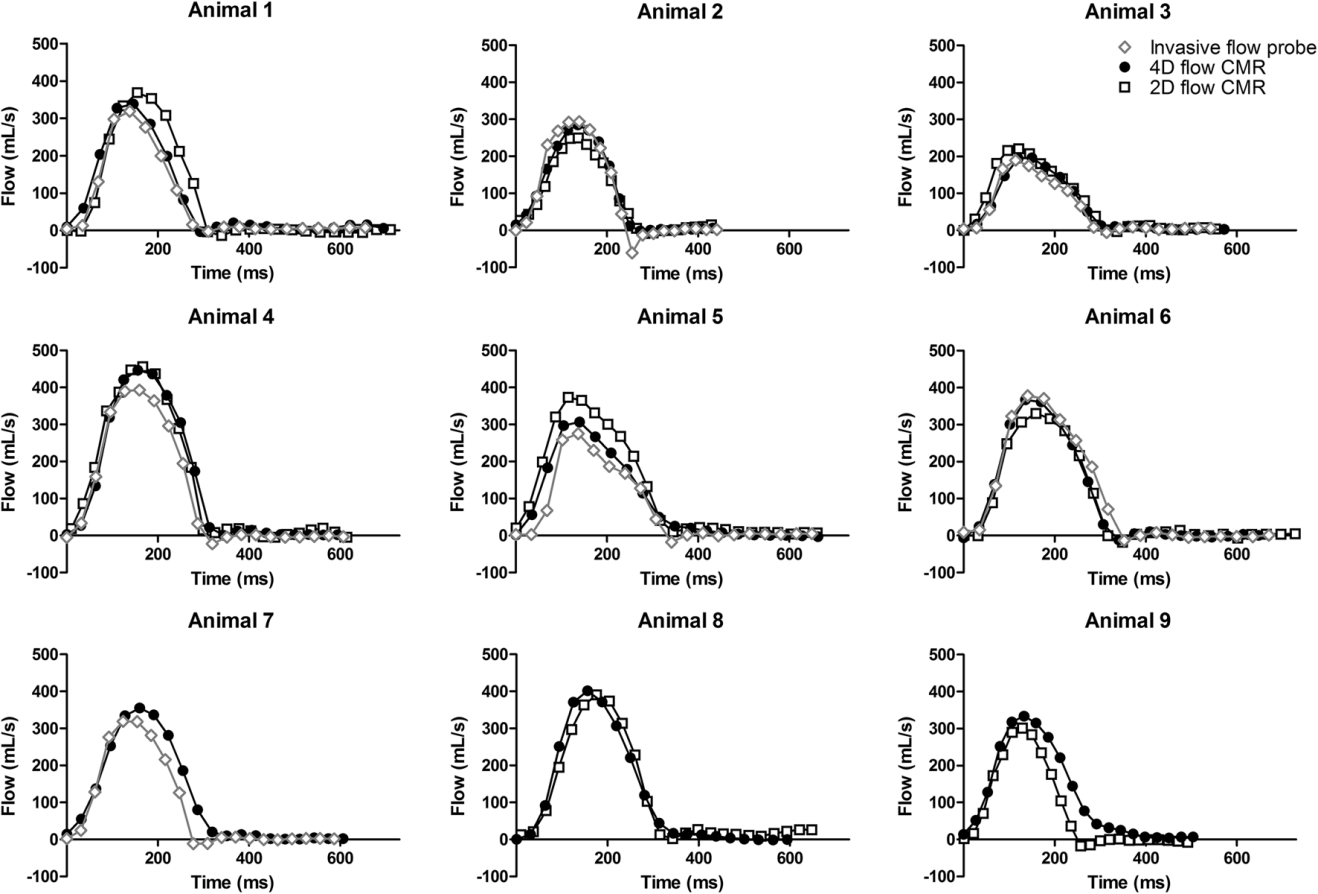
Individual aorta flow curves per heartbeat of the invasive flow probe, 4D flow CMR (measured at level of the aortic valve), and 2D flow CMR superimposed per animal. 2D flow CMR measurements of animal 7 and invasive flow probe measurements of animal 8 and 9 are missing due to technical problems

**Fig. 3:**
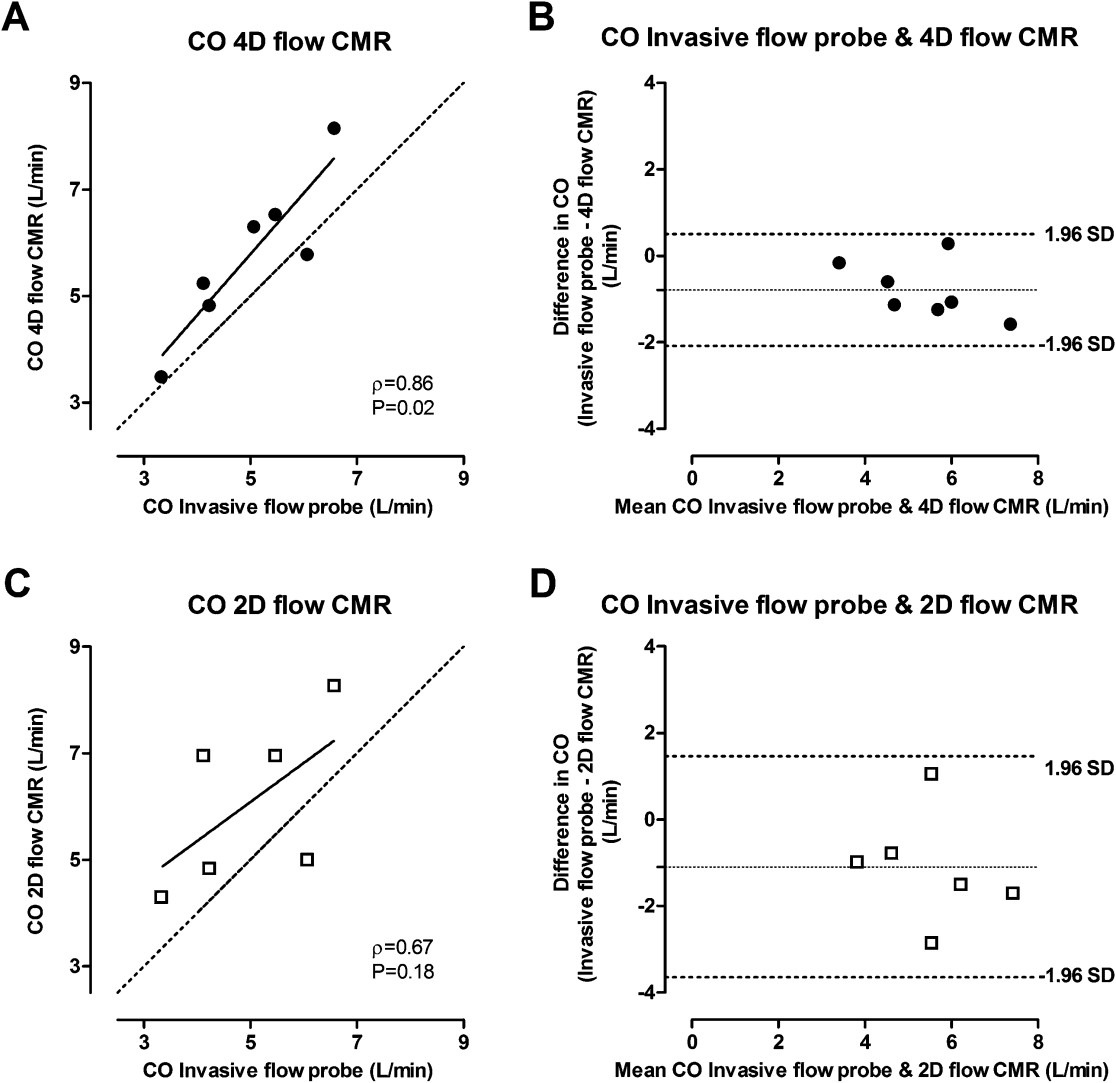
Comparison of cardiac output in the aorta measured by the invasive flow probe, 4D flow and 2D flow CMR Correlation between the invasive flow probe, 4D flow CMR (**a** n = 7), and 2D flow CMR (**c** n = 6) measurements of the aortic flow. Line of identity is indicated as the dotted line and Spearman’s rho (ρ) and *P* value are indicated in the legends (**a**, **c**). Bland–Altman plots of the cardiac output (CO), with the mean, 1.96 standard deviation (SD) and -1.96 SD lines indicated as the dotted lines. **b** invasive flow probe and 4D flow CMR measurements (n = 7), and **d** invasive flow probe and 2D flow CMR measurements (n = 6)

The shape of the individual aorta flow patterns measured by all three methods showed good agreement and individual flow curves per animal are depicted in Fig. 2. Indeed, the correlation between the CO measured by the invasive flow probe and 4D flow CMR was very strong (Spearman’s rho = 0.86 at the aortic valve level and 0.90 at the ascending aorta level) (Table 1 and Fig. 3). Relative to the invasive flow probe measurements, the flow measured by 4D flow CMR was overestimated by 0.8 L/min at the aortic valve level and by 0.6 L/min at the ascending aorta level (Bland–Altman, Table 1 and Fig. 3). The correlation between the invasive flow probe and 2D flow CMR and volumetric LV measurements were strong (2D flow CMR: Spearman’s rho = 0.67 and volumetric LV measurements: Spearman’s rho = 0.77). Relative to the invasive flow probe measurements, the flow measured by 2D flow CMR was overestimated by 1.1 L/min and by 1.3 L/min with the LV parameters (Bland–Altman, Table 1 and Fig. 3).

**Table 1:** Summary of flow measurements with the different methods

Method	Mean ± SD	n	Spearman’s rho^a^	Bland–Altman	
				Bias	± 1.96 SD
Invasive flow probe					
Heart rate (beats/min)	98 ± 16	7			
SV ascending aorta (mL/beat)	62 ± 15	7			
CO ascending aorta (L/min)	5.0 ± 1.2	7			
4D flow CMR					
Heart rate (beats/min)	98 ± 16	9			
SV aortic valve (mL/beat)	62 ± 14	9	0.86	− 8	− 23 to 6
CO aortic valve (L/min)	6.0 ± 1.4	9	0.86	− 0.8	− 2.1 to 0.5
SV ascending aorta (mL/beat)	56 ± 17	7	1.00	− 7	− 20 to 6
CO ascending aorta (L/min)	5.6 ± 1.5	7	0.90	− 0.6	− 1.8 to 0.6
SV MPA (mL/beat)	58 ± 15	9			
CO MPA (L/beat)	5.6 ± 1.3	9			
Qp:Qs	0.9 ± 0.1	9			
2D flow CMR					
Heart rate aortic valve (beats/min)	99 ± 20	8			
SV aortic valve (mL/beat)	62 ± 17	8	0.71	− 12	− 40 to 16
CO aortic valve (L/min)	5.9 ± 1.4	8	0.67	− 1.1	− 3.7 to 1.5
Heart rate MPA (beats/min)	102 ± 24	8			
SV MPA (mL/beat)	54 ± 21	8	0.60	− 3	− 32 to 27
CO MPA (L/min)	5.1 ± 1.5	8	0.81	− 0.4	− 2.7 to 2.0
Qp:Qs	0.9 ± 0.3	8	0.10	0.0	− 0.6 to 0.5
Left ventricular parameters					
Heart rate (beats/min)	103 ± 29	8			
End-diastolic volume (mL)	138 ± 32	8			
End-systolic volume (mL)	75 ± 25	8			
Ejaction fraction (%)	46 ± 9	8			
SV (mL/beat)	63 ± 16	8	0.94	− 12	− 28 to 4
CO (L/min)	6.3 ± 1.4	8	0.77	− 1.3	− 3.3 to 0.6

*MPA* main pulmonary artery, *SV* stroke volume, *CO* cardiac output

^a^Invasive flow probe measurements are taken as reference for the aortic flow measurements; 4D flow CMR measurements are taken as reference for the main pulmonary artery and Qp:Qs measurements

**Fig. 4:**
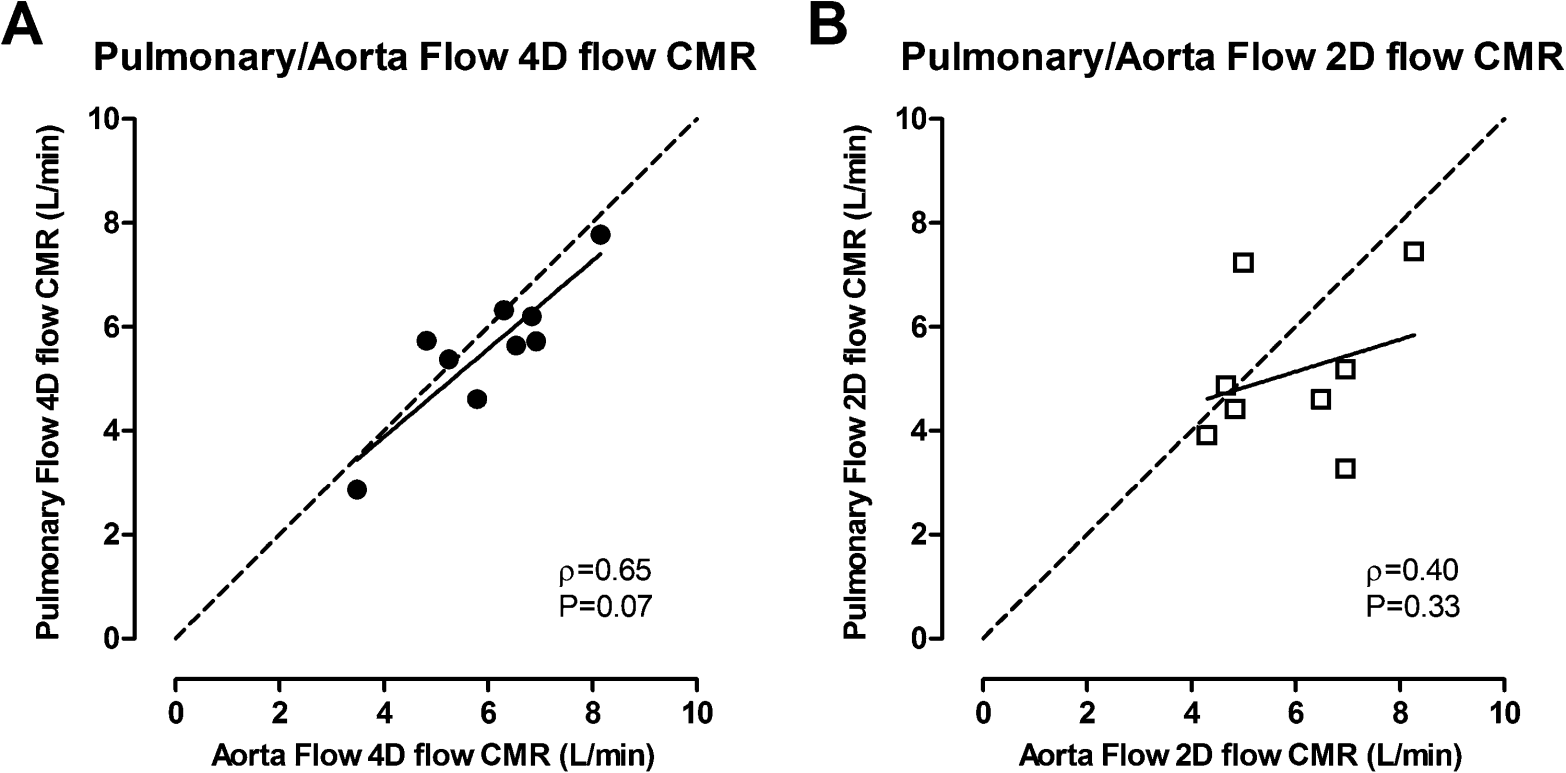
Comparison of pulmonary artery and aorta flow measured by 4D flow and 2D flow CMR Correlation between the aorta and pulmonary artery flow measured by 4D flow CMR (**a**, n = 9) and 2D flow CMR (**b**, n = 8). Line of identity is indicated as the dotted line. Spearman’s rho (ρ) and P-value are indicated in the legends

The correlation between the 4D flow CMR and 2D flow CMR MPA CO measurement was very strong (Spearman’s rho = 0.81) (Table 1). Relative to the 4D flow CMR measurements, the MPA CO measured by 2D flow CMR was underestimated by 0.4 L/min. Although the correlation between MPA and aorta flow at the aortic valve level as measured with 4D flow CMR only tended to be significant (Spearman’s rho = 0.65, P = 0.07), no correlation was found between MPA and aorta flow measured with 2D flow CMR (Spearman’s rho = 0.40, P = 0.33) (Fig. 4).

## Discussion

The main findings of this study were that (1) CO measured by 4D flow CMR showed a very strong correlation with invasively measured aortic flow, although there was an overestimation of the CO by 4D flow CMR of ~ 16%, (2) the correlation between 2D flow CMR and volumetric LV measurements in relation to invasively measured flow was less strong with a larger overestimation of the flow, and (3) the difference between MPA and aorta flow was much smaller with 4D flow compared to 2D flow CMR.

4D flow CMR is a relatively new technique but increasingly used in clinical practice due to its simplicity. The application of 4D flow CMR in the clinical setting provides many benefits as the technique is patient and operator friendly, less operator dependent and any flow in any plane can be selected retrospectively [8, 12, 22]. The latter is not the case for 2D flow CMR, in which flow measurements are limited to preselected planes [12, 22, 23]. Importantly, 4D flow CMR is in most cases more patient friendly than 2D flow CMR. One important benefit for the patients is that the sequence can be performed without breath holds (free breathing), which is not only more comfortable but will be an advantage in children and decompensated patients in which breath holds are mostly impossible [11, 12, 22]. In addition, data on flow and anatomic structures can be obtained simultaneously which can shorten the scan time for the patients, especially in patients with a difficult anatomy [11, 13, 22, 24]. However, it is important to note that there are some existing challenges with the 4D flow CMR technique such as long scan time, reliability of respiratory gating and lower resolution. Given the benefits and existing challenges, it is important to assess accuracy of 4D flow CMR measurements. Our data show that indeed, 4D flow CMR measurements correlate strongly with flow measured with an invasive flow probe, and overestimation of flow with this technique is less than measurements obtained using 2D flow CMR as well as compared to CO determined from volumetric LV measurements.

This is the first study to validate 4D flow CMR against both 2D flow CMR and invasively measured flow within the same animal, at the same time. Thus far, 4D flow CMR has only been validated against 2D flow CMR [13, 14] and ultrasound [12] measurements in humans. Although data are obtained in a relatively small group of animals, and magnetic interference of the metal in the flow probe could not be completely ruled out (two animals were excluded due to artifacts and in the other animals there were no noticeable imaging artifacts), both flow pattern and average flow showed a very strong correlation between invasively measured flow and 4D flow CMR. Although the invasively measured flow is considered the gold standard in validation studies, according to the manufacturer’s specifications, this technique has an absolute accuracy of 10%. Both 2D flow CMR and 4D flow CMR overestimated CO as compared to the invasively measured flow by 25% and 16% respectively. When comparing the individual flow patterns between the different techniques (Fig. 2) we do not see a consistent difference between the CMR and invasively measured flow, suggesting that there is no systematic bias. Consistent with data obtained in a canine model of acute thromboembolic pulmonary hypertension, 4D flow CMR resulted in slightly lower values of CO as compared to 2D flow CMR measurements [15], suggesting that determination of flow using 4D CMR is slightly more accurate than 2D CMR. The higher accuracy of 4D flow CMR vs 2D flow CMR can also be inferred from the comparison between aorta flow and MPA flow. Also here, there tended to be a correlation of the measurements with 4D flow CMR while no correlation was found with 2D flow CMR.

In conclusion, this study shows that aorta flow and pulmonary flow can be accurately and simultaneously measured by 4D flow CMR. This helps to further validate the quantitative reliability of this technique for implementation of 4D flow CMR in routine clinical practice.

## Electronic supplementary material

Below is the link to the electronic supplementary material.

Supplementary material 1 (MP4 322 kb)

Supplementary material 2 (MP4 443 kb)

## References

[1] Roest AA, Helbing WA, van der Wall EE, de Roos A (1999). Postoperative evaluation of congenital heart disease by magnetic resonance imaging. J Magn Reson Imaging.

[2] Uitterdijk A, Springeling T, van Kranenburg M, van Duin RW, Krabbendam-Peters I, Gorsse-Bakker C, Sneep S, van Haeren R, Verrijk R, van Geuns RJ, van der Giessen WJ, Markkula T, Duncker DJ, van Beusekom HM (2015). VEGF165A microsphere therapy for myocardial infarction suppresses acute cytokine release and increases microvascular density but does not improve cardiac function. Am J Physiol Heart Circ Physiol.

[3] Thomson LE, Crowley AL, Heitner JF, Cawley PJ, Weinsaft JW, Kim HW, Parker M, Judd RM, Harrison JK, Kim RJ (2008). Direct en face imaging of secundum atrial septal defects by velocity-encoded cardiovascular magnetic resonance in patients evaluated for possible transcatheter closure. Circ Cardiovasc Imaging.

[4] Valsangiacomo Buechel ER, Grosse-Wortmann L, Fratz S, Eichhorn J, Sarikouch S, Greil GF, Beerbaum P, Bucciarelli-Ducci C, Bonello B, Sieverding L, Schwitter J, Helbing WA (2015). Indications for cardiovascular magnetic resonance in children with congenital and acquired heart disease: an expert consensus paper of the Imaging Working Group of the AEPC and the Cardiovascular Magnetic Resonance Section of the EACVI. Cardiol Young.

[5] Vasanawala SS, Hanneman K, Alley MT, Hsiao A (2015). Congenital heart disease assessment with 4D flow MRI. J Magn Reson Imaging.

[6] Hanneman K, Kino A, Cheng JY, Alley MT, Vasanawala SS (2016). Assessment of the precision and reproducibility of ventricular volume, function, and mass measurements with ferumoxytol-enhanced 4D flow MRI. J Magn Reson Imaging.

[7] Hsiao A, Lustig M, Alley MT, Murphy M, Chan FP, Herfkens RJ, Vasanawala SS (2012). Rapid pediatric cardiac assessment of flow and ventricular volume with compressed sensing parallel imaging volumetric cine phase-contrast MRI. AJR Am J Roentgenol.

[8] Hanneman K, Sivagnanam M, Nguyen ET, Wald R, Greiser A, Crean AM, Ley S, Wintersperger BJ (2014). Magnetic resonance assessment of pulmonary (QP) to systemic (QS) flows using 4D phase-contrast imaging: pilot study comparison with standard through-plane 2D phase-contrast imaging. Acad Radiol.

[9] Hsiao A, Yousaf U, Alley MT, Lustig M, Chan FP, Newman B, Vasanawala SS (2015). Improved quantification and mapping of anomalous pulmonary venous flow with four-dimensional phase-contrast MRI and interactive streamline rendering. J Magn Reson Imaging.

[10] Valverde I, Simpson J, Schaeffter T, Beerbaum P (2010). 4D phase-contrast flow cardiovascular magnetic resonance: comprehensive quantification and visualization of flow dynamics in atrial septal defect and partial anomalous pulmonary venous return. Pediatr Cardiol.

[11] Chelu RG, Horowitz M, Sucha D, Kardys I, Ingremeau D, Vasanawala S, Nieman K, Paul JF, Hsiao A (2018). Evaluation of atrial septal defects with 4D flow MRI-multilevel and inter-reader reproducibility for quantification of shunt severity. MAGMA.

[12] Chelu RG, van den Bosch AE, van Kranenburg M, Hsiao A, van den Hoven AT, Ouhlous M, Budde RP, Beniest KM, Swart LE, Coenen A, Lubbers MM, Wielopolski PA, Vasanawala SS, Roos-Hesselink JW, Nieman K (2016). Qualitative grading of aortic regurgitation: a pilot study comparing CMR 4D flow and echocardiography. Int J Cardiovasc Imaging.

[13] Chelu RG, Wanambiro KW, Hsiao A, Swart LE, Voogd T, van den Hoven AT, van Kranenburg M, Coenen A, Boccalini S, Wielopolski PA, Vogel MW, Krestin GP, Vasanawala SS, Budde RPJ, Roos-Hesselink JW, Nieman K (2016). Cloud-processed 4D CMR flow imaging for pulmonary flow quantification. Eur J Radiol.

[14] Hsiao A, Alley MT, Massaband P, Herfkens RJ, Chan FP, Vasanawala SS (2011). Improved cardiovascular flow quantification with time-resolved volumetric phase-contrast MRI. Pediatr Radiol.

[15] Roldan-Alzate A, Frydrychowicz A, Johnson KM, Kellihan H, Chesler NC, Wieben O, Francois CJ (2014). Non-invasive assessment of cardiac function and pulmonary vascular resistance in an canine model of acute thromboembolic pulmonary hypertension using 4D flow cardiovascular magnetic resonance. J Cardiovasc Magn Reson.

[16] Stam K, van Duin RWB, Uitterdijk A, Cai Z, Duncker DJ, Merkus D (2018). Exercise facilitates early recognition of cardiac and vascular remodeling in chronic thromboembolic pulmonary hypertension in swine. Am J Physiol Heart Circ Physiol.

[17] Stam K, van Duin RWB, Uitterdijk A, Krabbendam-Peters I, Sorop O, Danser AHJ, Duncker DJ, Merkus D (2018) Pulmonary microvascular remodeling in chronic thrombo-embolic pulmonary hypertension. Am J Physiol Lung Cell Mol Physiol. 10.1152/ajplung.00043.2018 10.1152/ajplung.00043.2018 30260284

[18] De Wijs-Meijler DP, Stam K, van Duin RW, Verzijl A, Reiss IK, Duncker DJ, Merkus D (2016). Surgical placement of catheters for long-term cardiovascular exercise testing in swine. J Vis Exp.

[19] Duncker DJ, Stubenitsky R, Verdouw PD (1998). Role of adenosine in the regulation of coronary blood flow in swine at rest and during treadmill exercise. Am J Physiol.

[20] Stubenitsky R, Verdouw PD, Duncker DJ (1998). Autonomic control of cardiovascular performance and whole body O_2_ delivery and utilization in swine during treadmill exercise. Cardiovasc Res.

[21] Bland JM, Altman DG (1986). Statistical methods for assessing agreement between two methods of clinical measurement. Lancet.

[22] Dyverfeldt P, Bissell M, Barker AJ, Bolger AF, Carlhall CJ, Ebbers T, Francios CJ, Frydrychowicz A, Geiger J, Giese D, Hope MD, Kilner PJ, Kozerke S, Myerson S, Neubauer S, Wieben O, Markl M (2015). 4D flow cardiovascular magnetic resonance consensus statement. J Cardiovasc Magn Reson.

[23] Bollache E, van Ooij P, Powell A, Carr J, Markl M, Barker AJ (2016). Comparison of 4D flow and 2D velocity-encoded phase contrast MRI sequences for the evaluation of aortic hemodynamics. Int J Cardiovasc Imaging.

[24] Bock J, Töger J, Bidhult S, Markenroth Bloch K, Arvidsson P, Kanski M, Arheden H, Testud F, Greiser A, Heiberg E, Carlsson M Validation and reproducibility of cardiovascular 4D-flow MRI from two vendors using 2 × 2 parallel imaging acceleration in pulsatile flow phantom and in vivo with and without respiratory gating. Acta Radiol. 10.1177/0284185118784981 10.1177/0284185118784981 PMC6402051 30479136

